# Unique hole-accepting carbon-dots promoting selective carbon dioxide reduction nearly 100% to methanol by pure water

**DOI:** 10.1038/s41467-020-16227-3

**Published:** 2020-05-21

**Authors:** Yiou Wang, Xu Liu, Xiaoyu Han, Robert Godin, Jialu Chen, Wuzong Zhou, Chaoran Jiang, Jamie F. Thompson, K. Bayazit Mustafa, Stephen A. Shevlin, James R. Durrant, Zhengxiao Guo, Junwang Tang

**Affiliations:** 1 Department of Chemical Engineering, University College London, Torrington Place, London, WC1E 7JE UK; 2 Department of Chemistry, University College London, 20 Gordon Street, London, WC1H 0AJ UK; 3 Department of Chemistry, Imperial College London, Exhibition Road, London, SW7 2AZ UK; 4 Department of Chemistry, The University of British Columbia, Kelowna, BC V1V 1V7 Canada; 5 School of Chemistry, University of St. Andrews, St. Andrews, KY16 9ST UK; 6 Nanotechnology Research and Application Center, Sabancı University, Orta Mahallesi, Üniversite Cd. No: 27, 34956 Tuzla/İstanbul, Turkey; 7 Department of Chemistry, HKU-CAS Joint Laboratory on New Materials, The University of Hong Kong, Hong Kong, China; 8 Department of Mechanical Engineering, The University of Hong Kong, Hong Kong, China; 9 HKU Zhejiang Institute of Research and Innovation, The University of Hong Kong, Hangzhou, China; 10 Present Address: Chair for Photonics and Optoelectronics, Nano-Institute Munich, Ludwig-Maximilians-Universität München, Königinstr. 10, 80539 Munich, Germany; 11 Present Address: Department of Chemistry, The University of Manchester, Oxford Road, Manchester, M13 9PL UK

**Keywords:** Photocatalysis, Photocatalysis, Photocatalysis, Optical spectroscopy

## Abstract

Solar-driven CO_2_ reduction by abundant water to alcohols can supply sustainable liquid fuels and alleviate global warming. However, the sluggish water oxidation reaction has been hardly reported to be efficient and selective in CO_2_ conversion due to fast charge recombination. Here, using transient absorption spectroscopy, we demonstrate that microwave-synthesised carbon-dots (^m^CD) possess unique hole-accepting nature, prolonging the electron lifetime (*t*_50%_) of carbon nitride (CN) by six folds, favouring a six-electron product. ^m^CD-decorated CN stably produces stoichiometric oxygen and methanol from water and CO_2_ with nearly 100% selectivity to methanol and internal quantum efficiency of 2.1% in the visible region, further confirmed by isotopic labelling. Such ^m^CD rapidly extracts holes from CN and prevents the surface adsorption of methanol, favourably oxidising water over methanol and enhancing the selective CO_2_ reduction to alcohols. This work provides a unique strategy for efficient and highly selective CO_2_ reduction by water to high-value chemicals.

## Abstract (web summary)

Solar-driven CO2 reduction by abundant water to alcohols is hindered by the sluggish water oxidation reaction. Here, the authors demonstrate that the microwave-synthesized carbon-dots possess unique hole-accepting nature, allowing stoichiometric oxygen and methanol production from water and CO2 with nearly 100% selectivity to methanol.

## Introduction

The consumption of fossil fuels has been increasing the level of atmospheric CO_2_ and consequently causing imminent climate change^1–3^. To mitigate energy and environmental issues as well as generate sustainable fuels, people have devoted continuous efforts to reproducing natural photosynthesis to fix CO_2_ to sugar since the 1970s^4,5^. In an ideal photocatalytic CO_2_ conversion system, a semiconductor is excited by photons of appropriate energy and generates pairs of electrons and holes. These then transfer to the surface to reduce CO_2_ and oxidise water, competing with the undesired electron-hole recombination. Such recombination occurs at < µs timescale, much faster than the water oxidation by holes, which proceeds at ~1 s timescale^6^. Therefore, it remains challenging to reduce CO_2_ by pure water and benchmark systems mostly use sacrificial hole acceptors, instead of water, to rapidly scavenge holes and prolong the lifetime of photoelectrons^7,8^. Although the efficiencies of CO_2_ reduction have been increased by a few orders of magnitude in recent years, the majority of these systems are uneconomical and unsustainable^7,8^. In order to replace those sacrificial regents by abundant water to achieve sustainable CO_2_ conversion, one has to develop satisfactory semiconductors with long-lived charge carriers as well as co-catalysts to extract the photoholes, thus favouring the water oxidation reaction instead of charge recombination.

Carbon nitrides, which always comprise carbon, nitrogen and hydrogen instead of ideal structure C_3_N_4_ when prepared in the laboratory^9^ (denoted CN herein), is an emerging group of easy-to-tune organic semiconductors^10–12^ and has demonstrated uncommonly-observed long-lived charge carriers owing to its unique defective structure^13,14^. In the presence of reduction co-catalysts and visible-light illumination, CN has shown promising activities in hydrogen production and CO_2_ reduction due to its negatively-positioned conduction band (CB)^15–19^. However, there are only a few reports on water oxidation using CN, because its valence band (VB) is too close to the water oxidation potential as well as the inherent kinetic challenge of water oxidation^17,20^. Hence, an active co-catalyst for water oxidation is crucial to take advantage of long-lived charge carriers in CN. Carbon-dots (CD, < 10 nm in diameter), another emerging class of carbon nanomaterials with unique electronic structures, have recently been used as co-catalysts to enhance light absorption, decompose peroxide and more importantly store electrons for reduction reactions^17,21–24^. Although an electron-accepting CD co-catalyst prolongs the lifetime of electrons, it is associated with a loss of electrochemical potential of photogenerated electrons without accelerating the sluggish water oxidation. A better solution is to apply a hole-accepting CD to tackle the difficult water oxidation since electron-accepting co-catalysts with a less negative CB waste a portion of the reductive potential of electrons photogenerated in CN and do not thermodynamically favour proton reduction. However, limited evidence has been reported on CD for its function of selective charge carriers acceptance. On the other hand, such hole-accepting CD co-catalyst, albeit decisive for improved water oxidation, has been rarely observed in experiments until now^25–30^.

Methanol, a useful liquid hydrogen source with the convenience of storage and transport for fuel cells^31^, is a more desirable reduction product from CO_2_ compared to CO, methane and formic acid. However, the generation of methanol is a 6-electron process, hence requiring an exceptionally prolonged lifetime of charge carriers to allow for electrons accumulation. Moreover, methanol traps holes in ~10 ns on TiO_2,_ and its oxidation is thus kinetically much favoured over water oxidation (~1 s)^6^, making the continuous production of methanol a significant challenge. So far, stoichiometric production of methanol and oxygen from CO_2_ and water has hardly been reported with high selectivity (confirmed by isotopic labelling), long-term stability and satisfactory internal quantum yield (IQY) which is more reliable than product evolution rates (Supplementary Table 1)^32^, again due to the lack of suitable co-catalysts to selectively transfer holes towards water instead of methanol. Accordingly, in order to utilise water as a sustainable hole scavenger and selectively produce methanol from CO_2_, we need to design an ideal hole-accepting CD, which is mainly dependent upon our fundamental knowledge of photophysical processes^13^. Although transient absorption spectroscopy (TAS) plays an essential role in understanding the charge carrier dynamics of semiconductors for water splitting^13^, the spectroscopic characteristics in CO_2_ conversion processes are still unknown at ultrafast timescale, potentially hindering the rational development of target photocatalysts^33^.

Herein, using TAS investigation, we distinguish a unique species of CD as a hole acceptor in the ^m^CD/CN composite when fabricated by a scalable microwave method (^m^CD, graphite phase). CDs could also behave as an electron acceptor in the ^s^CD/CN composite where they are fabricated by a sonication-based method (^s^CD, amorphous). ^m^CD prolongs the lifetime of electrons in CN by a factor of four compared to ^s^CD, favouring the multi-electron reduction process. Remarkably, the ^m^CD/CN nanocomposite stably produces stoichiometric oxygen and methanol with near-unity selectivity, confirmed by ^13^C labelling, with an IQY of 2.1% at 420 nm. On the other hand, the ^s^CD/CN composite only generates CO, a two-electron product. Furthermore, the unique ^m^CD captures holes from electrons and prevents the adsorption of the reduction product methanol, hence favourably oxidising water instead of methanol and enhancing the selectivity of CO_2_ reduction to alcohols.

## Results

### Synthesis and characterisation of all photocatalysts

As discussed above, the widely reported electron-accepting co-catalysts with a less negative CB waste a portion of the reductive potential of electrons photogenerated in CN and do not promote the sluggish reaction between holes and water molecules. Hole-accepting co-catalysts can prolong the lifetime of photogenerated charges, preserve the reduction potential of photoelectrons on the CB and also turn water molecules to preferable products, e.g., O_2_, thus meeting our targeted ideal system for CO_2_ conversion. Therefore, an efficient hole-accepting co-catalyst is highly sought after for photon-driven CO_2_ reduction. We explored CD co-catalysts by synthesising two CD samples according to different recipes, and their properties were then investigated via extensive and thorough characterisations. Both carbon-dots were loaded to CN at their optimal concentrations: 3.5% for ^m^CD/CN and 3% for ^s^CD/CN, respectively.

The novel ^m^CD were synthesised via a modified 10-minute microwave method from urea and citric acid precursors, much faster than other reported methods for high-quality CD^17,24,28^. Then the purified ^m^CD were dissolved with dicyandiamide (DCDA, a precursor for CN) in DMF and stirred for ten hours at 60 °C before transferring into a ceramic crucible, followed by thermal polymerisation at 500 °C and exfoliation processes to obtain ^m^CD/CN. For comparison, the other carbon-dots reported as reduction co-catalysts were fabricated via an alkali-assisted sonication treatment of glucose precursor (^s^CD) and loaded to carbon nitride to form ^s^CD/CN^30^. Pristine CN was synthesised from a DCDA precursor followed by identical thermal oxidation etching^34^. Detailed nanocomposite photocatalyst preparation procedures and other characterisation methods are listed in the Method.

**Fig. 1:**
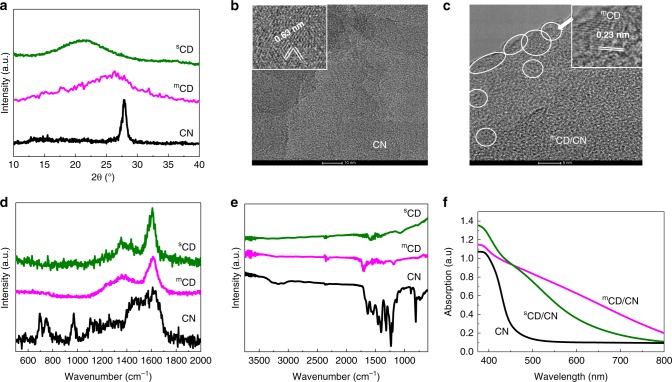
Characterisation of CN, ^m^CD/CN and ^s^CD/CN. **a** PXRD pattern of ^m^CD, ^s^CD and CN. **b** HRTEM images of CN. Scale bar: 10 nm. Inset: an enlarged image showing (110) crystal fringes of CN. **c** HRTEM images of ^m^CD/CN nanocomposite. Scale bar: 5 nm. ^m^CD are marked by circles. Inset: an enlarged image showing graphite superstructure of ^m^CD. **d** Raman, **e** Fourier transform infrared (FTIR) and **f** UV–vis spectra of ^m^CD, ^s^CD and CN. Source data are provided as a Source Data file.

Powder X‐ray diffraction (PXRD) patterns were measured (Fig. 1a) to obtain the crystallinity information of CN, ^m^CD/CN and ^s^CD/CN. At first glance, both carbon-dots exhibit poor crystalline patterns. However, ^m^CD shows a broadened (002) peak around 26.5° (3.36 Å)_,_ close to the graphite structure reported in the literature^35,36^. The weak and widened band of ^s^CD is positioned around 21.5°, far from a graphite-like pattern and may be assigned to an amorphous carbon compared to the former^34^. The synthesised CN polymer exhibits the typical diffraction signals of (002) and (100) planes at 27.4° (3.26 Å) and 13.0° (6.82 Å), respectively, indicating the successful formation of a heptazine-based polymer^37^. The co-existence and details of the structures of both ^m^CD and CN were examined by transmission electron microscopy (TEM). The as-prepared CN is graphene-like nanosheets (Fig. 1b) of several micrometres in diameter, with a very rough and crinkly surface due to the exfoliation of bulk CN (Supplementary Fig. 1a)^34^. High-resolution TEM (HRTEM) images of CN (Fig. 1b) show hexagonal lattice fringes with a characteristic d-spacing of 0.63 nm, which matches well with the (110) planes of CN (*a* = 1.277, *c* = 0.649 nm) ^34^. The diameter of the ^m^CD is 2–10 nm (Supplementary Fig. 1b). The CN matrix is coherently decorated by ^m^CDs, suggesting a strong interaction between the two phases (Fig. 1c and Supplementary Fig. 1d). The crystal structure of CN was relatively more sensitive to the electron beam irradiation and was therefore damaged after some irradiation, while that of ^m^CD remained intact. The ^m^CD synthesised here has a graphitic structure with characteristic d-spacing of about 0.23 nm (inset of Fig. 1c), which can be indexed to the (110) planes of a 2 × 2 × 1 hexagonal super unit cell of graphite (*a* = 0.46 and *c* = 0.67 nm). Notably, the high magnification images reveal that ^m^CD concentrate around the CN edges and boundaries, which potentially enhances change transfer process to water during photocatalysis. The ^s^CD synthesised from sonication of glucose under alkaline conditions are determined to be 15–20 nm in diameter, bigger compared to ^m^CD (Supplementary Fig. 1c). No long-range pattern of atomic positions was found, indicating an amorphous structure in line with the PXRD results. After the formation of a junction with CN, the crystalline structure of ^s^CD was again hardly found in TEM, likely due to its amorphous allotropic feature. Therefore, both XRD and TEM confirm the composite samples were successfully obtained, and the dominant structural difference between both carbon-dots is that ^m^CD shows a relatively more crystallised graphite structure while ^s^CD are not well crystallised, which might result in their different functions.

Raman spectroscopy was used to examine the backbone of the materials further. Both carbon-dots show relatively weak D-band around 1350 cm^−1^ and G-band around 1600 cm^−1^. ^m^CD have a smaller *I*_D_/*I*_G_ height ratio (0.399) compared to ^s^CD (0.495), indicative that ^s^CD exhibits a more significant deviation from a graphitic structure^38–40^, in agreement with the XRD and TEM results. Characteristic vibrations of the heptazine-based structure were found on CN, with the typical peaks in the regions of 1200–1700, 980 and 690 cm^−1^ (Fig. 1d) corresponding to disordered graphitic carbon-nitrogen vibrations, the symmetric N-breathing mode of heptazine and the in-plane bending, respectively^41,42^. Therefore, ^s^CD shows a more defective manner, likely amorphous carbon, while ^m^CD shows a more crystalised doped-graphite feature.

Complementary FTIR spectroscopy was carried out to identify the surface functional groups on the carbon structures. The vibrations in ^s^CD are mostly C–C vibrations at 1200 and 1450 cm^−1^ and show tiny C=O signals (Fig. 1e). In the absence of a significant amount of heteroatom dopants, ^s^CD still contains many defects according to the D-band in the Raman spectrum, confirming its amorphous carbon morphology. In addition to C–C features, ^m^CD also displayed stretching modes around 1250 and 1550 cm^−1^, attributable to C–N vibrations, and C=O stretching around 1730 cm^−1^, suggesting the existence of N and O moieties in graphite structure (Fig. 1e)^40,43^, consistent with the use of urea and citric acid precursors. The peaks around 2250–2550 cm^−1^ are attributed to adsorbed CO_2_ from the air. CN demonstrates a typical spectrum of carbon nitride^15^, exhibiting stretching and bending modes of nitrogen-containing heterocycles from 1000–1750 cm^−1^, and the broad feature at 3250 cm^−1^ is assigned to stretching modes of –NH– groups. X-ray photoelectron spectra (XPS, Supplementary Fig. 2) again confirmed the co-existence of N (NH_*x*_, C–N–C) and O species (C=O, C–O) in ^m^CD and ^m^CD/CN. After loading of both carbon-dots, the colours of samples changed obviously from pale yellow (CN) to dark brown (^m^CD/CN) or orange (^s^CD/CN). UV-visible spectra confirmed that both carbon-dots have a generally stronger absorption than CN in the visible region to 600–700 nm (Fig. 1f), commonly observed on carbon nanomaterials^38^. Taking into account the graphite backbone confirmed by XRD, TEM, Raman and FTIR, we can conclude that ^m^CD is N, O-doped graphite dots, which is more crystalline compared to the amorphous ^s^CD, hence shows somewhat increased visible-light absorption.

### Charge carrier dynamics of all samples

**Fig. 2:**
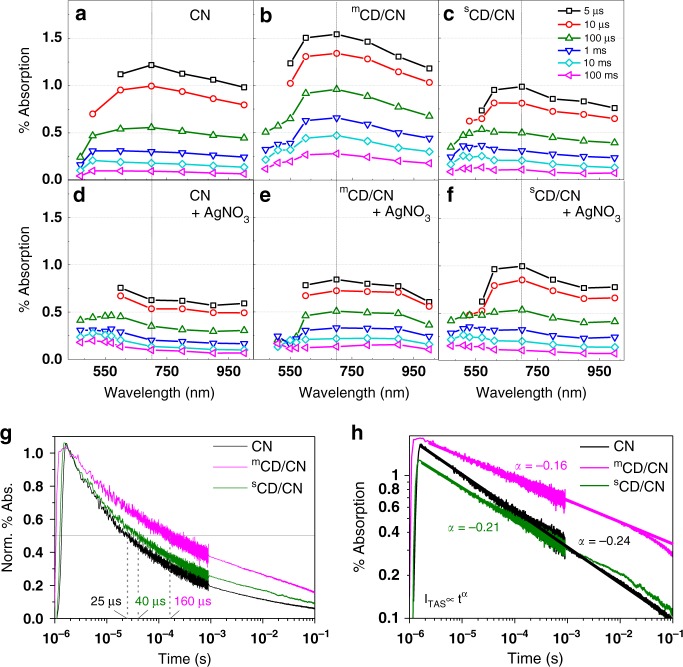
TAS measurements of CN, ^m^CD/CN and ^s^CD/CN. Diffuse reflectance TAS spectra for samples with (**a**–**c**) and without (**d**–**f**) 10 mM AgNO_3_. CN (**a**, **d**), ^m^CD/CN (**b**, **e**) and ^s^CD/CN (**c**, **f**) were dispersed in aqueous solution. The change of signal amplitude at 700 nm indicates it should be mainly assigned to the electron signal in CN and ^m^CD improves the charge separation on CN due to hole transfer from CN to ^m^CD. **g** μs-TAS decay kinetics of CN, ^m^CD/CN and ^s^CD/CN in water monitored at 700 nm and excited by pulsed 355 nm excitation (460 μJ/cm^2^) and **h** fitted α parameters indicated in the same color as the associated trace. Source data are provided as a Source Data file.

As discussed above, two major obstacles hindering sustainable light-driven CO_2_ reduction by water are (i) the intrinsic weak reduction potential of water and (ii) the lack of fundamental understandings of charge carrier dynamics of co-catalysts. Most of the reported CDs promote reduction process as an electron acceptor while very few are distinguished as an advantageous hole acceptor for efficient water oxidation. Therefore, to understand the role of different carbon-dots, we investigated the charge transfer kinetics of CN, ^m^CD/CN and ^s^CD/CN samples after synthesis and characterisation. TAS, a powerful technique to elucidate the charge carrier dynamics of photocatalysts, was used to experimentally determine the electron-hole dynamics of ^m^CD/CN and ^s^CD/CN nanocomposite at µs-s timescales. Photoexcitation of CN alone yields a broad absorption spanning from 450 to 1000 nm, which peaks near 700 nm (Fig. 2a, dashed line). In order to determine the nature of the excited states, we first measured the TAS spectra of CN with and without Ag^+^ ions, a well-known efficient electron scavenger which can yield long-lived holes. Kinetic traces probed at 510 and 700 nm (Supplementary Fig. 3a, b) show that in the presence of Ag^+^ electron scavenger the amplitude at 700 nm decreases but the 510 nm signal enhances and features a longer-lived species. Therefore, the signal observed at 510 nm is mainly assigned to photogenerated holes in CN and the broad photoinduced signal observed at 700 nm is assigned to photogenerated electrons in CN, similar to previous observations on CN^13^.

Turning to the CD/CN nanocomposites, the TAS spectra of ^m^CD/CN (Fig. 2b) resemble that of the CN rather than the isolated ^m^CD (Supplementary Fig. 3c). Moreover, the change in collected probe light is on the order of 1% for both the ^m^CD/CN and CN, while it is on the order of 0.005% for ^m^CD. These considerations indicate that TAS predominantly probes CN, and the main feature observed for the ^m^CD/CN nanocomposite remains that of electrons localised in CN. Compared to the results with CN, the amplitude of the 700 nm feature (electron signals in CN) is higher (increasing from 1.25 to 1.5%) in the case of ^m^CD/CN (Fig. 2b), suggesting that more efficient charge separation increases the number of long-lived electrons localised on CN at these timescales. The increased electron signal in CN indicates that holes were efficiently extracted from CN to ^m^CD. In contrast, ^s^CD/CN shows a smaller signal amplitude than pure CN (decreasing from 1.25 to 0.95% at 700 nm) throughout the probed window, inferring electron transfer from CN to ^s^CD. The addition of AgNO_3_ does not affect the signal (Fig. 2c,f), suggesting that electrons were already effectively extracted from CN by ^s^CD.

The half-life time (for an initial time *t*_0_ = 2 µs) of the signal observed at 700 nm increases over 6-fold from 25 µs (CN) to 160 µs (^m^CD/CN) (Fig. 2g), indicating suppressed electron-hole recombination due to charge separation across the ^m^CD/CN junction. A long-lived shoulder near 550 nm for ^s^CD/CN was also noted, in line with the 510 nm feature of holes residing in CN. A slight increase in t_50%_ (from 25 to 40 µs) compared to bare CN (Fig. 2g) was attributed to the separation of charges by ^s^CD. These observations are consistent with the hypothesis of the extraction of electrons from CN by ^s^CD. The μs-TAS decay at 700 nm is well represented by a power law of the form *I* ∝ *t*^−*α*^, as evidenced by the linear decay on a log–log plot, indicating a trapping-detrapping dynamics (Fig. 2h)^13^. Briefly, we have identified the desired hole-accepting ^m^CD co-catalyst in the ^m^CD/CN composite. Different functions of two CDs could be seen for charge separation, and the specific CD/CN interaction can influence the direction of charge transfer.

### Photocatalytic CO_2_ conversion to methanol

After identification of the different structures and physical functions of these junctions, the activity of the photocatalysts for reduction of CO_2_ in pure water was evaluated under 1 bar of CO_2_ at room temperature under visible-light irradiation (*λ* > 420 nm; see Supplementary Figs. 6–8 for raw data and calibration curve).

**Fig. 3:**
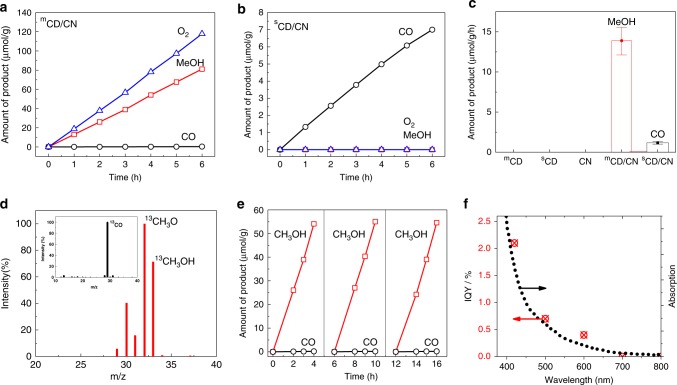
Photocatalytic CO_2_ conversion to methanol. The photocatalytic activity of **a** ^m^CD/CN and **b** ^s^CD/CN measured under visible light (*λ* > 420 nm). **c** Control experiments on ^m^CD, ^s^CD, CN, ^m^CD/CN and ^s^CD/CN. Error bar: ^m^CD/CN 13.9 ± 1.7 µmol/g/h, ^s^CD/CN 1.2 ± 0.2 µmol/g/h. **d** Mass spectra of the product ^13^CH_3_OH from ^13^CO_2_ photoconversion by the ^m^CD/CN photocatalyst. Inset: the mass spectra of the ^13^CO from ^13^CO_2_ photoconversion over the ^m^CD/CN photocatalyst. **e** Consecutive three runs of CO_2_ photoconversion to methanol on ^m^CD/CN under visible light. **f** IQY of ^m^CD/CN measured at atmospheric pressure under nearly one sun irradiation condition. Source data are provided as a Source Data file.

The ^m^CD/CN nanocomposite, which contained hole-accepting ^m^CD, exhibits a remarkably enhanced activity towards the production of methanol and oxygen from CO_2_ photoreduction with water as the only electron donor (Fig. 3a). The concentration of ^m^CD was optimised to 3.5% wt (Supplementary Fig. 5a), presumably, to balance light absorption and reaction sites for water oxidation since further increases in the ^m^CD concentration lowers the photoactivity. The yields of both methanol and CO on ^m^CD (3.5)/CN increase nearly linearly over time (Supplementary Fig. 5b) and the average production rates are 13.9 and 0.05 µmol/g/h_,_ showing a ~99.6 ± 0.2% (calculated from three consecutive runs) selectivity towards methanol. Moreover, O_2_ is evolved as the only oxidation product, and the evolved amount increases linearly with the reaction time, similar to the production profile of CO and methanol (Supplementary Fig. 5b). The ratio of O_2_ to methanol is about 1.45:1, very close to the expected stoichiometric ratio of 1.5:1 when assuming all generated electrons reduce CO_2_ to methanol and all holes oxidise water to O_2_. Neither H_2_ nor oxidation products other than O_2_ were detected. The evolution of stoichiometric O_2_ again confirms the significance of the unique function of hole-accepting ^m^CD.

On the contrary, ^s^CD as a reduction co-catalyst was also optimised to a 3% loading on CN to form ^s^CD/CN, which only produces the two-electron product CO instead of the six-electron product methanol, together with negligible O_2_ gas (Fig. 3b). This is not surprising because CN itself can hardly produce oxygen in the absence of hole-accepting co-catalysts and ^s^CD here is an electron acceptor as proved by our TAS results, which facilitate CO_2_ reduction to CO. A similar CD/g-C_3_N_4_ junction was previously reported for CO_2_ reduction to CO and others^30^. A tiny amount of generated O_2_ might also be dissolved in water, consumed via back reactions or below the detection limit of GC. In total, the photocatalytic activity of ^m^CD/CN is ca. 12 times higher than ^s^CD/CN for CO_2_ reduction under identical experimental conditions.

Several control experiments were also performed (Fig. 3c): (i) without a CO_2_ feed (for ^m^CD/CN nanocomposite), (ii) in the absence of a photocatalyst and (iii) with a lack of light. Under these conditions, the production of both CO and methanol were negligible, indicating that the CN and ^m^CD could not be decomposed to the products under irradiation. Also, ^m^CD or pure CN showed nearly zero activity. The physical mixture of ^m^CD and CN was also tested and showed no evident activity compared to pure CN, which also supports that a strong interaction between ^m^CD/CN was only prepared in situ by microwave heating and is crucial for CO_2_ photoreduction.

Isotopic measurement is crucial to avoid misleading information because inevitable organic impurities in carbon-based catalysts can possibly be oxidised to hydrocarbons (e.g., methanol or other organic products). To further confirm the conversion of CO_2_, the photoreduction of ^13^C-labeled CO_2_ was conducted over the ^m^CD/CN photocatalyst. Dominant peaks of ^13^CO (*m*/*z* = 29) and ^13^CH_3_OH^+^ (*m*/*z* = 33) were observed at *m*/*z* = 29, 32 and 33, which were assigned to ^13^CO, ^13^CH_3_OH^+^ and fragments of ^13^CH_3_O^+^ produced during the MS measurement (Fig. 3d). The evidence indicates that the evolved products originate from the photoreduction of ^13^CO_2_.

Three consecutive runs were then carried out over the ^m^CD/CN photocatalyst (Fig. 3e) to investigate its stability. The CO and methanol evolution rates did not show noticeable changes, indicating the excellent stability of the nanocomposite. For comparison with other reported systems, quantum yield (QY) is the more preferable and more reliable metric to evaluate one material’s property rather than activity, since the total production rate is highly dependent on the experimental conditions^9^. The IQY (Fig. 3f) of the optimised ^m^CD/CN photocatalyst was measured to be 2.1% at *λ* = 420 nm, 0.7% at *λ* = 500 nm and 0.4% at even *λ* = 600 nm under 1 bar conditions at room temperature, which is over an order of magnitude higher than that recently reported for CN (0.076% at 420 nm)^30^. The IQYs at longer wavelengths might be due to the light absorption of ^m^CD or ^m^CD/CN interfacial states as CN could not harvest 500–600 nm photons by itself. Long-lived TAS signal attributed to charges is observed in the ^m^CD/CN composite but not with CN alone under 600 nm excitation (Supplementary Fig. 4). Therefore, water has been successfully used as the sole electron source in the photoreduction of CO_2,_ and for the first time, methanol was produced with nearly unity selectivity in one step under visible-light.

To further demonstrate the improved properties of the ^m^CD/CN composite, we prepared and tested photoelectrodes. The photocurrent of ^m^CD/CN photoanode is approximately twice higher than that of the CN photoanode (Supplementary Fig. 5c), demonstrating the improved separation efficiency of photoexcited charge carriers in ^m^CD/CN and nature of hole acceptor of ^m^CD, which agrees with the results of CO_2_ photoconversion. To better understand the photophysical characteristics of photoinduced charge carriers in photocatalysts, the ns-level time-resolved fluorescence of ^m^CD/CN and CN were measured (Supplementary Fig. 5d). The ^m^CD/CN presents slower decay kinetics compared with pure CN, namely, the photogenerated charge carriers in ^m^CD/CN live longer than those generated by CN. The average lifetimes of the carriers (*τ*_avg_) were determined to be ~15.3 and 13.3 ns for ^m^CD/CN and CN according to the fitting calculation, respectively, supporting better electron transfer and higher charge separation efficiency in ^m^CD/CN.

It seems ideal that methanol, a highly desirable product from CO_2_ conversion, has been produced with near-unity selectivity and remains intact. This is particularly remarkable because methanol is among the most commonly used holes scavengers for hydrogen production from water as it is easily and rapidly oxidised (~10 ns timescale), as mentioned above. How can methanol be conserved as an inert product in a photocatalytic system which generates holes continuously? To elucidate the high selectivity of the ^m^CD/CN junction for H_2_O oxidation and methanol production, we employed theoretical calculations of adsorption energy based on Density Functional Theory (DFT). The calculations were carried out using the Vienna ab-initio Simulation Package (VASP)^44^. To mimic the structure of the ^m^CD, the model (Supplementary Fig. 9c) consisting of a coronene (Supplementary Fig. 9a) with a pyrene (Supplementary Fig. 9b) on the top was placed in a 20 × 20 × 10 Å^3^ box. Two nano-flakes were symmetrically mass-centred with a relaxed interlayer distance of 3.37 Å, consistent with the XRD and HRTEM observation (Fig. 1a,c). This model has been proven effective to mimic the CD (Supplementary Fig. 9a–c)^39^. For the CN, we have adopted the structures used in the previous calculations (Supplementary Fig. 9d,e)^22^. Detailed computational settings are illustrated in the SI.

**Fig. 4:**
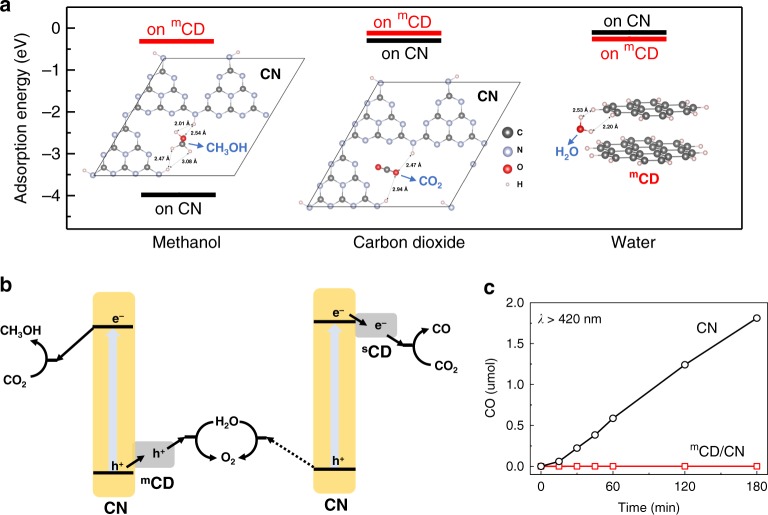
The fundamental understanding of the high selectivity to methanol. **a** Adsorption energies and the most stable configurations of CO_2_ and CH_3_OH on CN and H_2_O on ^m^CD. **b** Schematic diagram of photocatalytic CO_2_ reduction by the ^m^CD/CN and ^s^CD/CN. The band bending between catalysts and water/CO_2_ is not drawn. **c** The CH_3_OH oxidation test on CN and ^m^CD/CN under visible light. Source data are provided as a Source Data file.

The adsorption energy, *E*_ad_, was calculated according to

1 $$E_{{\mathrm{ad}}} = E_{{\mathrm{total}}} - \left( {E_{{\mathrm{adsorbate}}} + E_{{\mathrm{adsorbent}}}} \right)$$

where *E*_total_, *E*_adsorbate_ and *E*_adsorbent_ represent the energies of the absorbing system, the adsorbate and the adsorbent at equilibrium configurations, respectively. According to the calculated adsorption energy of the most stable configurations (Supplementary Tables 3 and 5), both CO_2_ and more importantly CH_3_OH bind more favourably to CN rather than ^m^CD (Fig. 4a left and middle). On the contrary, H_2_O molecules prefer to be absorbed on the surface of ^m^CD instead of CN (Fig. 4a right). According to the TAS results and calculations of E_ad_ on ^m^CD/CN, CN maintains electrons and adsorb CO_2_, while ^m^CD selectively accepts holes and absorbs H_2_O. Hence for the ^m^CD/CN composite, CO_2_ is reduced by photoelectrons on CN and photoholes on ^m^CD are preferably used to oxidise H_2_O instead of methanol. Based on the above results, a pathway of visible-light-driven CO_2_ photoconversion on ^m^CD/CN has been proposed in Fig. 4b. Electron-hole pairs are generated in CN under visible-light excitation. Photoelectrons accumulate on the surface of CN where CO_2_ reduction to CH_3_OH occurs, while photoholes transfer from CN to the surface of ^m^CD to oxidise H_2_O into O_2_. On the other hand, for ^s^CD/CN, CO_2_ reduction takes place on electron-accepting ^s^CD to produce CO while the holes on CN relatively slowly oxidise H_2_O molecules or oxidise methanol if produced. Thus, CO could come from both direct reduction of CO_2_ and the subsequent oxidation of methanol, which is difficult to distinguish in this case.

To confirm the compatibility of methanol with ^m^CD/CN to rationalise the high selectivity to methanol experimentally, we directly tested the oxidation of methanol by ^m^CD/CN and CN. Figure 4c shows that under visible-light, methanol can be readily oxidised to CO by photoholes of pure CN, which might be the reason why there is no methanol produced by ^s^CD/CN. Interestingly, in the ^m^CD/CN system, no CO was detected, consistent with the theoretical calculations that methanol could not be readily adsorbed on the surface of ^m^CD where holes accumulate, and photooxidation of water occurs. Therefore, the ^m^CD possesses the unique functions of selectively accepting photoholes as well as selectively absorbing water instead of methanol. Hence, the desired hole-accepting carbon-dots co-catalysts have been successfully developed to utilise water as the only electron donor in CO_2_ conversion and guarantees near unity selectivity of methanol. The origin of its superior performance has been rationalised by modelling as well as experiments.

## Discussion

For ^s^CD, its amorphous carbon nature indicates a mixture of sp^2^ and *sp*^3^ species^45^. Hence the charge transfer is less efficient than pure sp^2^ hybrid of the graphite structure (^m^CD), which partially explains why the ^m^CD shows a larger enhancement of lifetime of charges. Holes on CN could not intrinsically oxidise water in the absence of co-catalysts. The activity of O_2_ production is thus negligible and CO_2_ reduction is not efficient due to fast charge recombination in ^s^CD/CN and poor hole activity. On the other hand, ^m^CD/CN favours electron accumulation on the surface of CN due to efficient hole transfer to ^m^CD, which facilitates a multi-electron reduction process to produce methanol instead of CO. Therefore, this study demonstrates the superiority of the design of hole-accepting co-catalysts ^m^CD to the widely reported electron acceptor ^s^CD in promoting the reaction of CO_2_ reduction to higher-value chemicals.

To summarise, through TAS investigations over six orders of magnitude in time on carbon-based nano architectures, we observe that ^s^CD work as an electron acceptor in ^s^CD/CN, while ^m^CD serves as a notable hole acceptor in ^m^CD/CN after excitation. Charge separation across the CD/CN interface increases the lifetime of the charge carriers from 25 µs (CN) to 160 µs (^m^CD/CN) or only 40 µs (^s^CD/CN) and enhances participation in subsequent CO_2_ conversion. The differences in photophysical functions of CDs result in the differences in both conversion efficiency and more importantly, selectivity. ^s^CD/CN junction converts CO_2_ into CO while the ^m^CD/CN junction could unprecedentedly and selectively reduce CO_2_ to methanol, which has been further confirmed by ^13^C labelling with nearly unity selectivity. Furthermore, the ^m^CD/CN composite is c.a. 12  times more active than ^s^CD/CN for CO_2_ conversion observed under the same experimental conditions.

Such ^m^CD show more favourable water adsorption yet unfavourable methanol adsorption compared to CN, thus facilitating selective oxidation of water to O_2_ while avoiding unproductive oxidation of the photoreduction product, methanol. Therefore, the uncommon ^m^CD co-catalyst is a crucial reason for such high selectivity of CO_2_ reduction to methanol using water as the only reductant. Overall, electrons reach the surface of CN and possess enough chemical potential to reduce CO_2_, together with protons, to produce methanol with an exceptional 99.6 ± 0.2% selectivity and high IQY of 2.1% at 420 nm and benchmark IQY at 500 and 600 nm (0.7 and 0.4%, respectively). The unique function of hole-accepting carbon-dots should not only excite broad interest in selective photocatalytic oxidation in organic synthesis and environmental purification but also be potentially useful for promoting charge transfer in photovoltaics, photoelectrochemical devices and light-emitting diodes. This work paves a metal-free avenue to the sustainable production of methanol, which is among the most promising hydrogen sources for fuel cells.

## Methods

### Materials preparations

All chemical reagents were analytical grade and were used without further purification. Citric acid, *N*,*N*-dimethylformamide (DMF), urea, dicyandiamide, methanol, dichloromethane were purchased from Sigma-Aldrich Company Ltd. Deionised water used in all the experiments has a resistivity of 18.1 MΩ·cm.

### CN Nanosheets

Dicyandiamide (2 g) in a closed crucible was heated at 500 °C for 4 h in static air with a ramp rate of 5 °C/min. The yellow solid agglomerates were milled into powders using an agate mortar. 0.5 g of this as-prepared powders were placed on a ceramic plate and heated at 500 °C for 4 h in air with a ramp rate of 10 °C/min. Then, the CN nanosheets with light yellow colour were finally obtained. This CN nanosheets sample was named CN.

### Carbon-dots via microwave method (^m^CD)

The citric acid (3 g, 15.6 mmol) and urea (1 g, 16.7 mmol) were added into 8 ml deionised water in a beaker and vigorously stirred to form a transparent solution. This beaker containing solution was then moved into the microwave oven and heated for 10 min under the power mode of 600 W. During the reaction, the solution changed from a colourless liquid to a dark brown porous solid, which indicates the CD was finally produced^46^. The solid product was then put into an oven and dried at 80 °C for 10 h to remove the small residual molecules. The suspension of the crude CD was purified in a centrifuge at 8000 rpm for 1 h to remove large or agglomerated particles. The final brown aqueous solution was eluted with a mixture of methanol and dichloromethane at ratios of 1:2 and 1:1 (v/v) to obtain ^m^CD. At last, the resulting ^m^CD were dried into solid powders.

### ^m^CD/CN nanocomposite

Dicyandiamide (2 g) and x mg of ^m^CD obtained above (*x* = 30, 50, 70 and 90, equal to w.t. 1.5, 2.5, 3.5, and 4.5% to dicyandiamide precursor) were added to 10 mL of DMF and stirred for 1 h; then the liquid was dried at 60 °C for 10 h to completely evaporate DMF. The resulting mixture was placed into a crucible with a cover and annealed at 500 °C in the air for 4 h with a ramping rate of 5 °C/min. The brown solid was washed with DI water and dried at 80 °C, then milled into powders with an agate mortar. A concentration of 0.5 g of powders was placed on a ceramic plate and heated at 500 °C for 4 h in air with a ramp rate of 10 °C/min. Finally, the ^m^CD/CN nanocomposite was obtained. The samples were denoted as ^m^CD(n)/CN (*n* = 1.5, 2.5, 3.5 and 4.5) by the weight percentage of carbon-dots in the precursor.

### Carbon-dots via sonication method (^s^CD)

Glucose (18 g) was dissolved in 200 mL 0.5 M NaOH solution before ultrasonication for 2 h^30^. A dark brown solution was gradually formed, implying the successful production of the CD. The crude solution was neutralised to pH = 7 using 0.1 M HCl. The suspension of the crude CD was washed by deionised water and purified in a centrifuge at 8000 rpm for 1 h to remove large or agglomerated particles. At last, the resulting ^s^CD were dried into solid powders.

### ^s^CD/CN nanocomposite

Pure CN (1 g) was dispersed in 200 mL of 0.5 M HCl and ultrasonicated for 4 h at room temperature before repeated wash with deionised water and centrifugation to remove excess HCl^30^. Lastly, the protonated CN was dried in an oven overnight at 70 °C and then ground into powder. Next, 1 g protonated CN and an optimal 3% w.t. of CD sample was dispersed in 100 mL of deionised water and sonicated for 15 min and stirred vigorously at room temperature for 30 min. The mixture was then subjected to hydrothermal treatment in a Teflon-sealed autoclave with stirring at 120 °C for 4 h to form the ^s^CD/CN nanocomposite.

### Characterisations

The morphologies and structures of the samples were characterised by HRTEM using an FEI Titan Themis microscope, operated at 200 kV. UV/Vis spectra were carried out on a Shimadzu UV/Vis 2550 spectrophotometer with an integrating sphere device at RT and transformed to the absorption spectra according to the Kubelka-Munk relation. XPS measurements were recorded by using a Thermo Scientific XPS spectrometer. Time-resolved fluorescence emission spectra were carried out at room temperature with a Fluorescence spectrophotometer (Edinburgh Instruments, FLSP-920) monitoring at 470 nm (excitation wavelength of 330 nm). The X-ray diffraction (XRD) patterns were measured by a PANalytical X’pert MPD Pro diffractometer operated at 40 kV and 40 mA using Ni-filtered Cu-Kα irradiation (Wavelength 1.5406 Å). ATR-FTIR spectroscopy was performed using a Perkin-Elmer 1605 FTIR spectrometer in the wavenumber range of 500–4000 cm−1 with a resolution of 0.5 cm^−1^. Raman spectroscopic measurements were performed on a Renishaw InVia Raman Microscope, using a 325 nm excitation laser and a wavenumber range of 100–2000 cm^−1^.

### Photocatalysis measurement

Before the photocatalytic reduction of CO_2_, 10 mg photocatalyst and 10 mL water were added into a septum-sealed borosilicate glass reactor with a volume of 140 mL. Then, the reactor was purged with CO_2_ for the photoreduction experiment. A 300 W Xe lamp (Newport) was utilised as a light source, and the light output power was measured by a Newport 918-D calibrated photodetector. During the reaction, the products were analysed by GC (Varian GC-450) with a thermal conductivity detector (TCD, connected to a molecular sieve column) and a flame ionisation detector (FID, connected to a CP-SIL 5CB capillary column) containing a methanizer equipment. Ar gas was used as the GC carrier gas.

For the isotope-trace experiment, the same photocatalytic process was applied except ^13^CO_2_ (^13^C 99%, Sigma-Aldrich) was used as the feed gas. The products containing C-isotope were analysed by GC–MS (Shimadzu QP-2010SE) with a molecular sieve 5 Å capillary column (for CO) or a Rxi-624Sil MS capillary column (for methanol). He gas was used as a carrier gas during the measurement.

The CH_3_OH oxidation conditions: 10 ml H_2_O, 0.12 µmol MeOH, 10 mg ^m^CD/CN photocatalyst, 300 W Xenon lamp irradiation with 420 nm long-pass filter in 1 bar Argon atmosphere.

### Fabrication of film electrodes and electrochemical measurements

A total of 10 mg photocatalyst and 10 μL Nafion solution (5 wt%) were dispersed in a water/methanol mixture (1 mL, 1:1 v/v). After sonication treatment for at least 2 h, the mixture formed a homogeneous catalyst colloid. Before the measurements, the catalyst colloid (200 μL) was deposited on an FTO conductive glass with an area of ~1 cm^2^ to form the working electrode. The photocurrent was measured using three electrodes at 1 V bias voltage, of which Ag/AgCl and Pt net electrodes were used as the reference and counter electrodes, respectively. The electrolyte was 1 M NaSO_4_ aqueous solution degassed with Ar.

### Calculation of internal quantum efficiency

The internal quantum yields for CD/CN photocatalyst was measured using the same experimental setup as the photocatalysis measurement, with a bandpass filter (*λ* = 420, 500, or 600 nm). The average intensity of irradiation after the 420, 500, and 600 nm bandpass filter was 221, 347^,^ and 398 µW/cm^2^, respectively (calculated by five points measured in the beam).

2 $${\mathrm{Absorption}}_{\mathrm{t}} = 100{\mathrm{\% }} \times \frac{{ - \Delta T_{\mathrm{t}}}}{{T_0}} = 100{\mathrm{\% }} \times \frac{{ - \left( {T_{\mathrm{t}} - T_0} \right)}}{{T_0}} = - 100{\mathrm{\% }} \times \left( {10^{ - A} - 1} \right)$$

The absorbed light density is 110.9, 134.5, and 132.7 µW/cm^2^ (for λ=420, 500, or 600 nm, respectively). For the photocatalytic reaction, 10 mg CD/CN photocatalysts were dispersed into 10 ml DI water. The measurement was carried out under a 300 W Xe lamp with the bandpass filter for 4 h. The amount of CO and CH_3_OH is 0.00083 and 0.235, 0.00025, and 0.114 µmol, and 0.00010 and 0.074 µmol for 420, 500, and 600 nm bandpass filters, respectively. Also, the irradiation area of the reactor is ~12 cm^2^. Thus, the IQYs at 420, 500, and 600 nm is c.a. 2.1, 0.7, and 0.4%, respectively.

The internal quantum yields are defined by the following equation

3 $${\mathrm{IQY}} = \frac{{{\mathrm{number}}\,{\mathrm{of}}\,{\mathrm{reacted}}\,{\mathrm{electron}}}}{{{\mathrm{number}}\,{\mathrm{of}}\,{\mathrm{absorbed}}\,{\mathrm{photon}}}} \times 100{\mathrm{\% }}$$

Two electrons are consumed per CO molecule evolved, and six electrons are consumed per CH_3_OH molecule evolved according to reaction (3) or (4).

4 $${\mathrm{CO}}_2 + 2{\mathrm{e}}^ - + 2{\mathrm{H}}^ + \to {\mathrm{CO}} + {\mathrm{H}}_2{\mathrm{O}}$$

5 $${\mathrm{CO}}_2 + 6{\mathrm{e}}^ - + 6{\mathrm{H}}^ + \to {\mathrm{CH}}_3{\mathrm{OH}} + {\mathrm{H}}_2{\mathrm{O}}$$

As a result, the internal quantum efficiency can be estimated by the equation:

6 $${\mathrm{IQY}} = \frac{{{{N}}_{{\mathrm{CO}}} \times 2 \times N_{\mathrm{A}} + {{N}}_{{\mathrm{MeOH}}} \times 6 \times N_{\mathrm{A}}}}{{{{H}}_{\mathrm{a}} \times {{A}} \times \frac{\lambda }{{hc}} \times t}}$$

where *N*_CO_ is the amount of CO after 4 h reaction, *N*_MeOH_ is the amount of CH_3_OH after 4 h reaction, *N*_A_ is the Avogadro’s number, *H*_a_ is the average intensity of absorbed light, obtained by the subtraction of the transmitted intensity from the incident intensity. *A* is the irradiation area (12 cm^2^), *h* is the Planck’s constant, *c* is the speed of light, *λ* is the wavelength of the incident light, *t* is the time.

## Supplementary information

Supplementary Information

## Data Availability

The source data underlying Figs. 1a,d–f, 2a–h, 3a–f and 4a,c and Supplementary Figs 2a–d, 3a–c, 4 and 5a–d are provided as a Source Data file.

## Source data

Source Data
