# Supplementary material for: Unique hole-accepting carbon-dots promoting selective carbon dioxide reduction nearly 100% to methanol by pure water
Source: Nat Commun. 2020 May 21;11:2531. doi: 10.1038/s41467-020-16227-3 (PMC7242399; doi:10.1038/s41467-020-16227-3)
Supplement: Supplementary file 1 — Supplementary Information [file 41467_2020_16227_MOESM1_ESM.pdf]

Supplementary Information

Unique hole-accepting carbon-dots promoting selective carbon dioxide reduction nearly 100% to methanol by pure water

Wang et al.

Unique hole-accepting carbon-dots promoting selective carbon dioxide reduction nearly 100% to methanol by pure water

Yiou Wang[1]†, Xu Liu[1]†, Xiaoyu Han[2], Robert Godin[3, 4,] *, Jialu Chen[4], Wuzong Zhou[4], Chaoran Jiang[1], Jamie F. Thompson[3], Mustafa, K. Bayazit[1, 6], Stephen A. Shevlin[2], James R. Durrant[3,] Zhengxiao Guo[2,7,8,9] * Junwang Tang[1,] *

Author affiliations

1. Department of Chemical Engineering, University College London, Torrington Place, London, WC1E 7JE, UK.
2. Department of Chemistry, University College London, 20 Gordon Street, London, WC1H 0AJ, UK.
3. Department of Chemistry, Imperial College London, Exhibition Road, London, SW7 2AZ, UK.
4. Department of Chemistry, The University of British Columbia, Kelowna, BC, V1V 1V7, Canada.
5. School of Chemistry, University of St. Andrews, St. Andrews, KY16 9ST, UK.
6. Nanotechnology Research and Application Center, Sabancı University, Orta Mahallesi, Üniversite Cd. No:27, 34956 Tuzla/İstanbul Turkey.
7. Department of Chemistry, HKU-CAS Joint Laboratory on New Materials, The University of Hong Kong, Hong Kong, China.
8. Department of Mechanical Engineering, The University of Hong Kong, Hong Kong, China.
9. HKU Zhejiang Institute of Research and Innovation, The University of Hong Kong, Hangzhou, China.

Corresponding authors

*junwang.tang@ucl.ac.uk. robert.godin@ubc.ca. zxguo@hku.hk.

Supplementary Table 1. A literature review of CO_2 reduction to MeOH under visible light in aqueous environments with reported quantum yields.

	Photocatalyst	IQY@420nm if not specified	^{13}C isotopic	O_2 gas measurement	Selectivity/%	Reference
1	^{m}CD/CN	2.1 (420nm), 0.7 (500nm), 0.4 (600nm)	√	√	99.6	This work
2	anatase–brookite TiO_2 composite	0.0717	×	×		[2]
3	NiO/InTaO4	0.063	×	×		[3]
4	mesoporous flake g-C_3N_4	0.18	×	×	58.3	[4]
5	rGO–CuO	1.3 (visible LED)	×	√		[5]
6	graphene− TiO_2	2.3 (lamp in the middle of reactor)	×	×		[6]
7	$BiVO_4$ (0.2g)	0.22 (visible)<br>0.24 (full sepectrum)	×	√		[7]
8	Carbon nitride-CdS QD	0.91@435 (apparent QY)	×	×	73	[8]
9	$In_2O_{3-x}(OH)_y$	0.19@250ºC	×	×	50	[9]

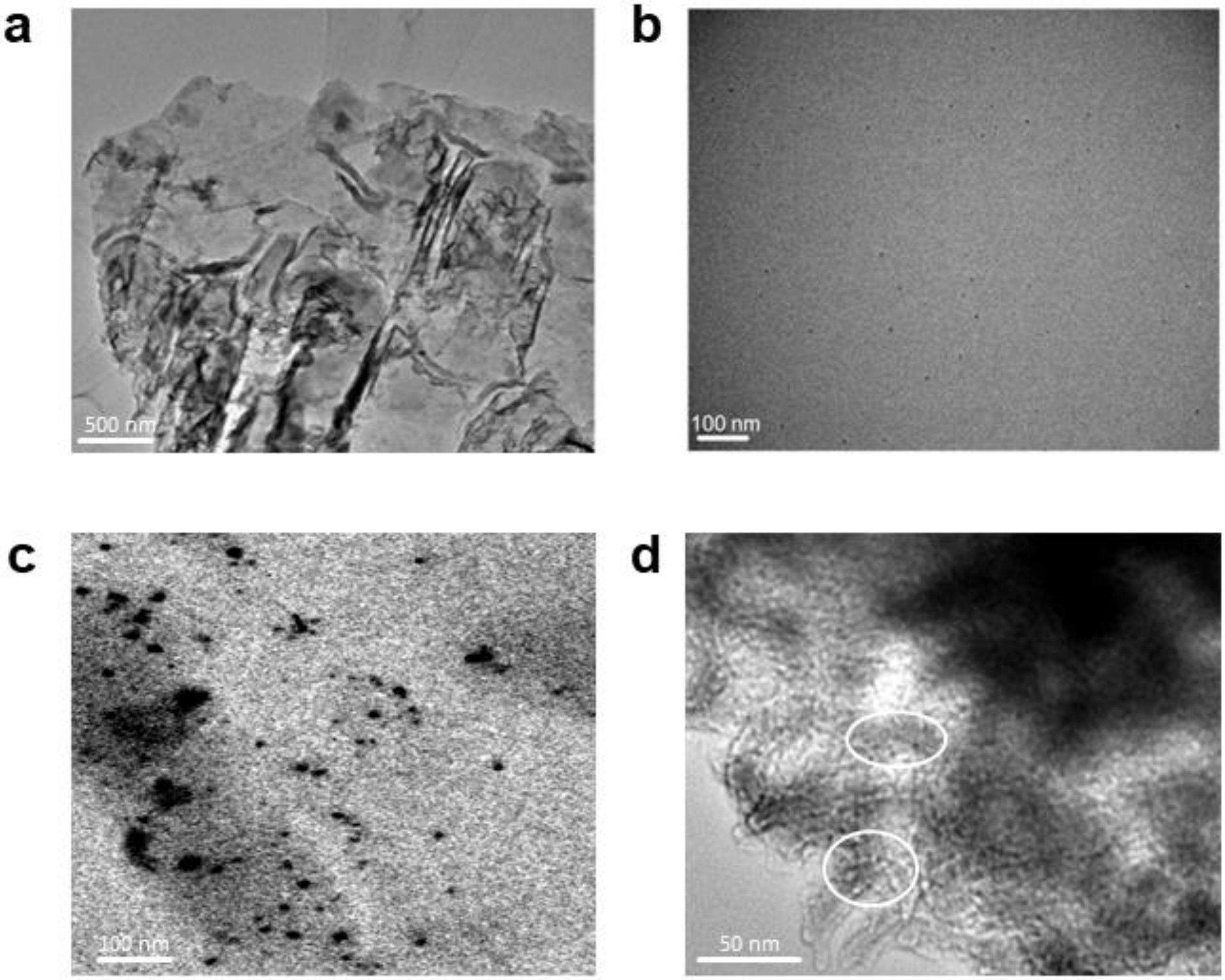

Supplementary Figure 1. Low magnification TEM image of (a) CN, (b) **^{m}**CD prepared by the microwave-assisted method, (c) **^{s}**CD prepared by the sonication method and (d) nanocomposite of ^{m}CD (marked by circles)/CN.

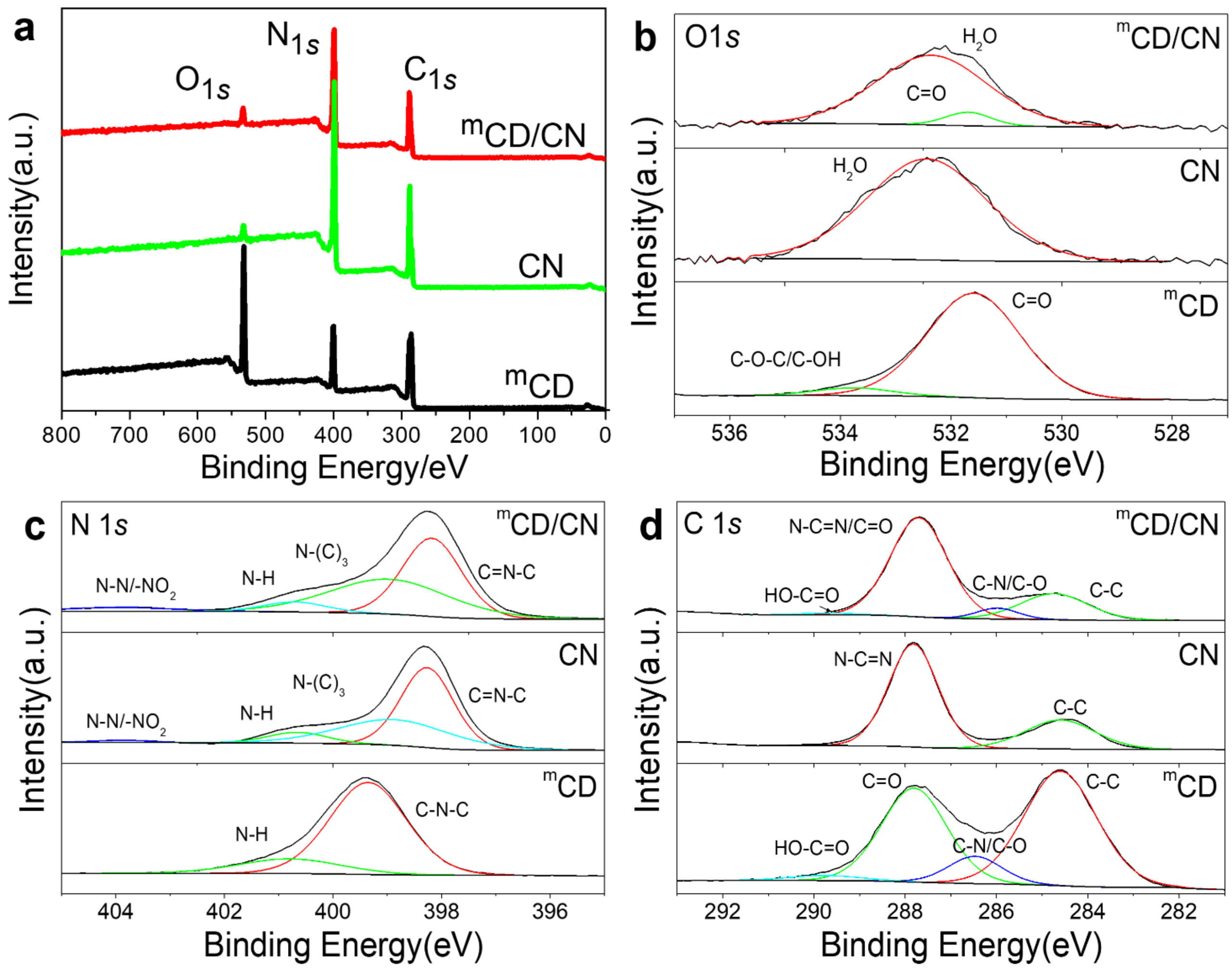

Supplementary Figure 2. (a) The survey XPS spectra of CN, ^{m}CD and ^{m}CD/CN. (b) The C1s XPS spectra, (c) the N1s XPS spectra, and (d) the O1s XPS spectra of CN, ^{m}CD and ^{m}CD/CN. Source data are provided as a Source Data file.

Supplementary Table 2. Surface compositions of C,N,O from XPS for the samples used in this study (Supplementary Figure 2.a)

Sample	C(at%)	C(wt%)	N(at%)	N(wt%)	O(at%)	O(wt%)
^{m}CD	61.99	56.00	11.86	12.50	26.15	31.50
CN	48.41	44.44	49.26	52.67	2.33	2.89
^{m}CD/CN	49.19	45.09	47.17	50.46	3.64	4.45

Supplementary Methods

Transient absorption spectroscopy.

TAS data were acquired on home-built setups. Samples were purged with argon to remove oxygen. 355 nm or 600 nm laser excitation was generated from an Nd:YAG laser (OPOTEK Opolette 355 II, 7 ns pulse width). The 355 nm excitation fluence was set to 460 µJ/cm^2 for the heterogeneous CN, ^{s}CD/CN and ^{m}CD/CN dispersions and 680 µJ/cm^2 for the homogeneous ^{m}CD only sample. 200 µJ/cm^2 of 600 nm excitation was used to see the effect of visible light excitation. The probe light was generated from a quartz halogen lamp (Bentham IL1). Long pass filters (Comar Instruments) were placed between the lamp and sample to minimize short-wavelength irradiation of the sample. A 5 cm path length cuvette filled with DI water was also placed in the beam path as an IR filter to avoid heating effects. A long pass filter positioned between the sample and a monochromator was used to block the scattered laser light. The probe wavelength was selected by the monochromator and the light relayed to a Si photodiode detector (Hamamatsu S3071). Data on the sub-ms timescale were conditioned by an electronic amplifier box (Costronics) and recorded on an oscilloscope. Data on the ms timescale were simultaneously recorded on a National Instruments DAQ card. Acquisitions were triggered by a photodiode (Thorlabs DET10A) exposed to laser scatter. Data from at least 32 laser pulses were acquired and processed using home-built software written in the Labview environment to obtain the kinetic traces presented.

A typical transmission setup was used in the case of the homogeneous CD samples (0.6 mg/mL aqueous solution) where the change in transmitted light intensity is measured. A diffuse reflectance setup was used in the case of the scattering CN ^{s}CD/CN and ^{m}CD/CN samples (2 mg/mL aqueous dispersion). Here the diffusively reflected light is collected by a 2″ diameter, 2″ focal length lens and relayed to the monochromator, and the change in reflected light taken as the change in absorption (i.e. Absorption + Reflectance = 1 – Transmission = constant).

We convert the absorbance signal to the relative change in the detected light intensity (in transmission mode) by using:

$$\Delta \mathrm{OD_t} = -\log\left(\frac{T_t}{T_0}\right); 10^{-\Delta \mathrm{OD_t}} = \frac{T_t}{T_0} \quad (1)$$

$$10^{-\Delta \mathrm{OD_t}} - 1 = \frac{T_\mathrm{t}}{T_0} - \frac{T_0}{T_0} = \frac{T_\mathrm{t} - T_0}{T_0} \quad (2)$$

$$\%\mathrm{Absorption_t} = 100 \times \frac{-\Delta T_\mathrm{t}}{T_0} = 100 \times \frac{-(T_\mathrm{t} - T_0)}{T_0} = -100 \times (10^{-\Delta \mathrm{OD_t}} - 1) \quad (3)$$

ΔOD is the change in absorbance (in absorbance units), T is the transmission (in decimal units), *%Absorption* is the relative change in absorbance (in percentile), and the subscripts 0 and t indicate either the initial value or the value at time t.

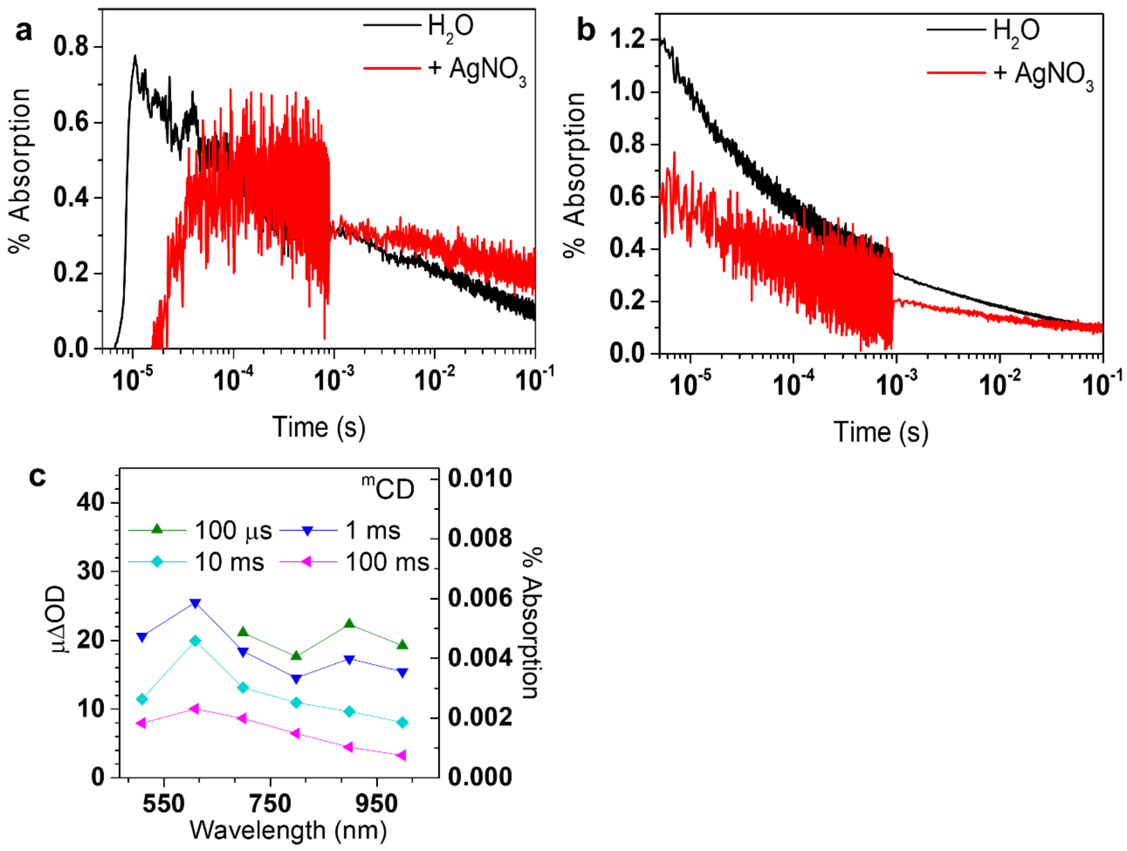

Supplementary Figure 3. TAS kinetics probed at 510 nm (a) and 700 nm (b) of CN dispersed in H_2O (black) and 10 mM $AgNO_3$ aqueous solution (red). (c) Transmission TAS spectra of **^{m}**CD (0.6 mg/mL) in aqueous solution. Source data are provided as a Source Data file.

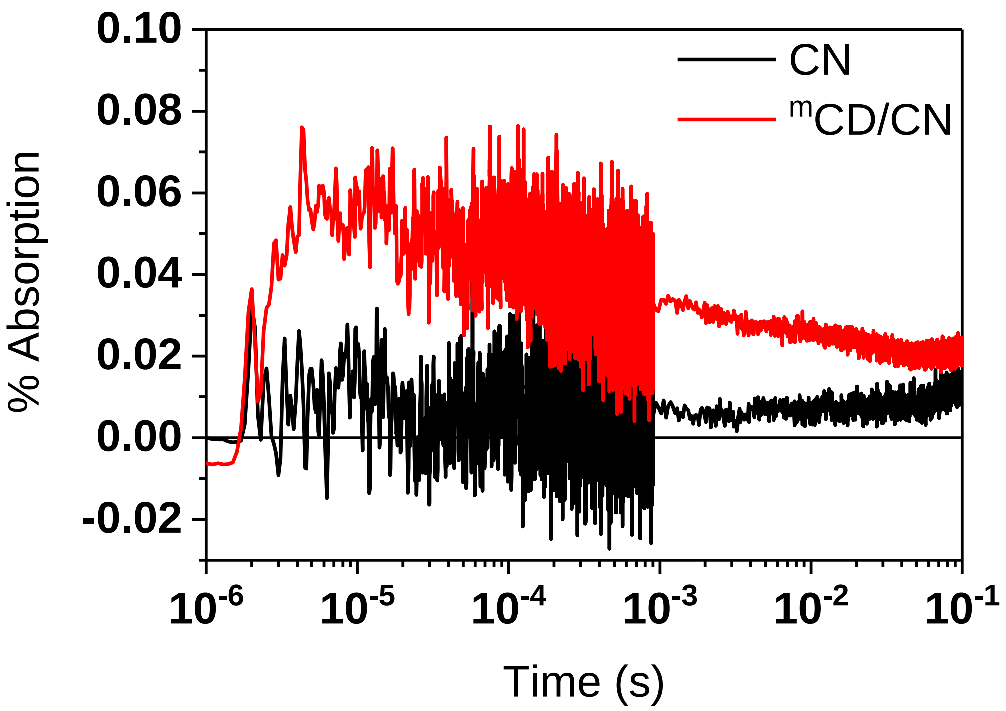

Supplementary Figure 4. TAS kinetics probed at 900 nm of CN and ^{m}CD/CN aqueous dispersions (2 mg/mL) under 600 nm excitation. Source data are provided as a Source Data file.

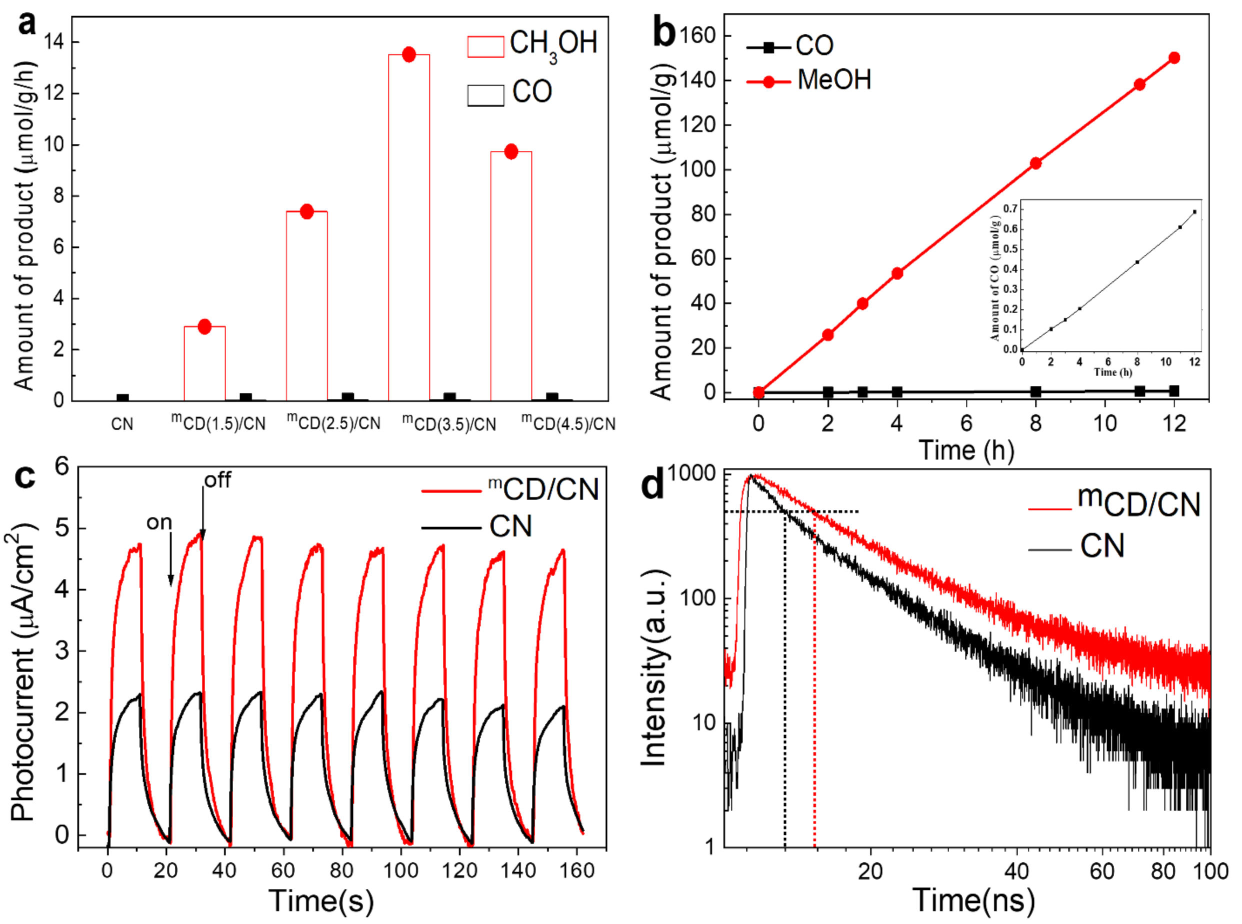

Supplementary Figure 5. (a) Photocatalytic CO and CH_3OH evolution over samples of CN, ^{m}CD(1.5)/CN, ^{m}CD(2.5)/CN, ^{m}CD(3.5)/CN and ^{m}CD (4.5)/CN. The values of 1.5, 2.5, 3.5 and 4.5 are corresponding to the mass ratio between ^{m}CDs and dicyandiamide precursor in percentage (1.5%, 2.5%, 3.5% and 4.5%, respectively) prior to the synthesis. (b) Stability test under visible light irradiation (c) Photocurrent profiles of ^{m}CD/CN and CN measured using three electrodes at 1 V bias voltage under AM 1.5 100 mW/cm^2; (d) Time-resolved fluorescence decay trace of ^{m}CD/CN and CN monitored at 470 nm (excitation wavelength of 330 nm). Source data are provided as a Source Data file.

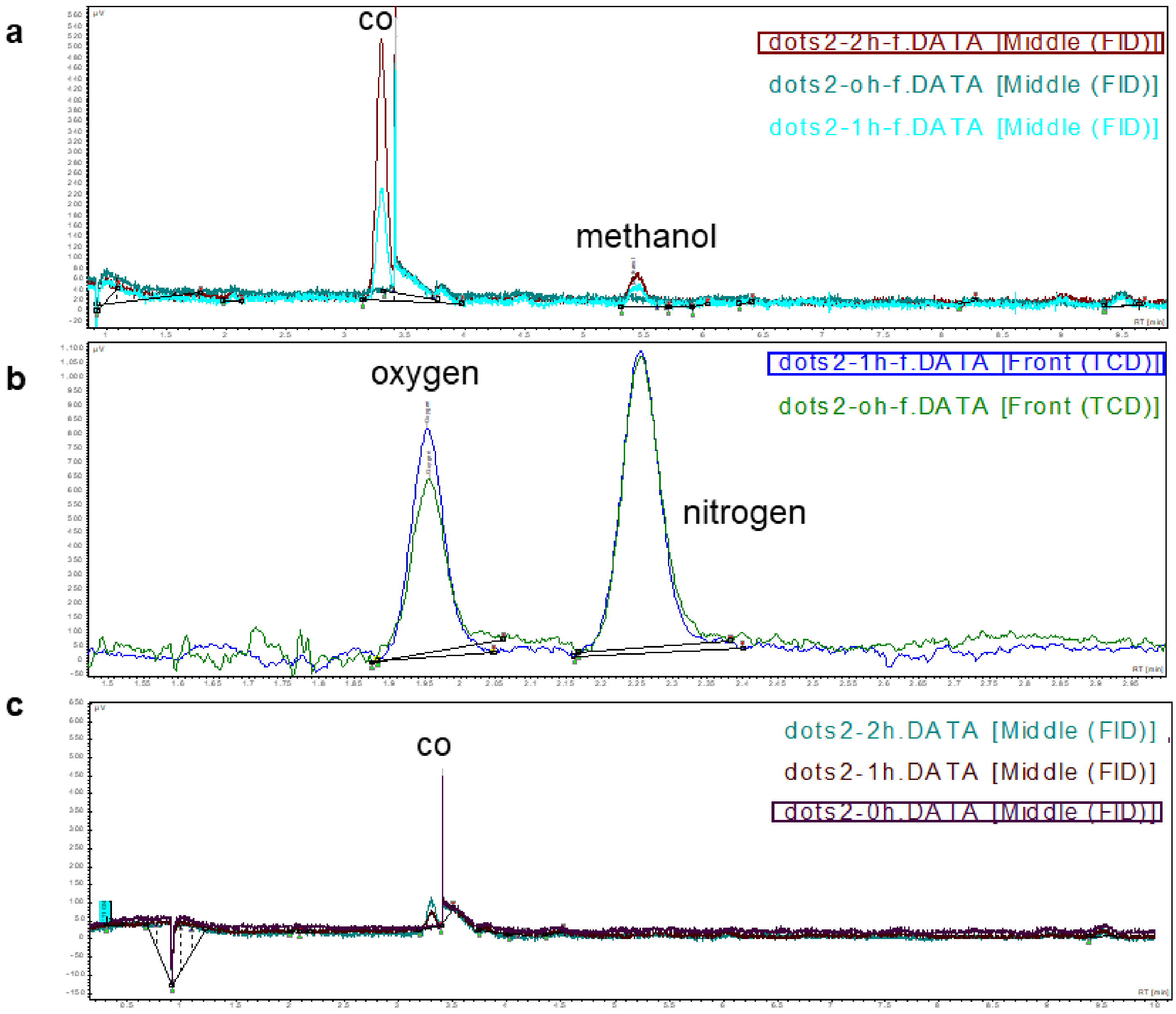

Supplementary Figure 6. Raw data: GC-traces of products during the reaction. Curves with different colours indicate the measurements after 0h, 1h and 2h of light irradiation, which shows the increasing of products. (a) CO and methanol measured by FID on ^{m}CD/CN. The retention time for CO is ~3.4 min. The retention time for methanol is ~5.4 min. 0h: dark cyan, 1h: cyan, and 2h: red. (b) Oxygen and nitrogen measured by TCD on ^{m}CD/CN. The retention time for oxygen is ~1.95 min. The retention time for nitrogen is ~2.25 min. 0h: olive, 1h: blue. (c) CO measured on ^{s}CD/CN.0h: wine, 1h: light wine, 2h: dark cyan.

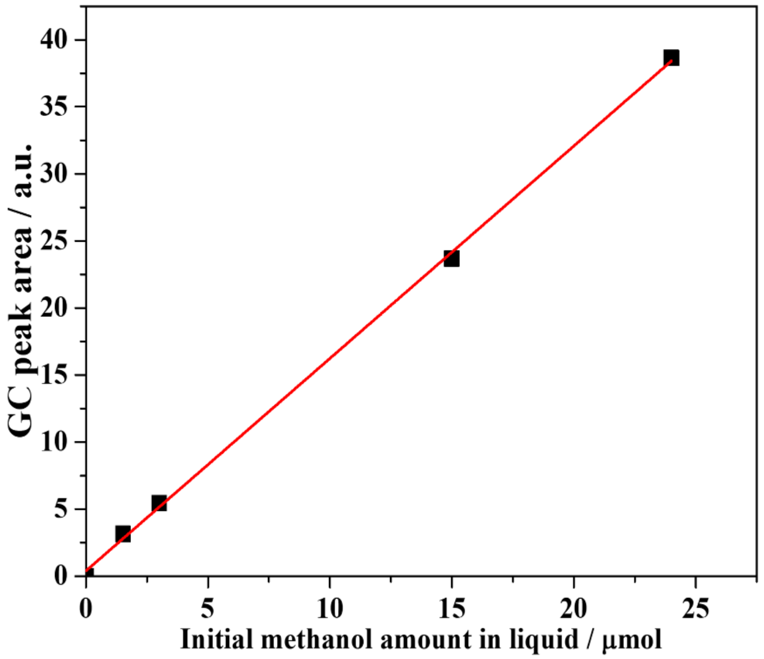

Supplementary Figure 7. Calibration curve of different amount of methanol in water of experimental GC (Varian 450) under working condition.

Supplementary Note:

The calibration method is similar to our previous report[1]. The amount of alcohol was determined by measuring the amount of methanol in the headspace of the reactor. According to Raoult's law, in a sealed system, liquid and vapour phases reach equilibrium thermodynamically at a certain temperature and pressure. Various 10 ml aqueous solutions containing different concentrations of methanol were first sealed in a gas-tight reactor with argon as the balance gas. The solution was stirred under light irradiation for 20 min. Then, 1 ml of the headspace vapour was analysed by gas chromatography.

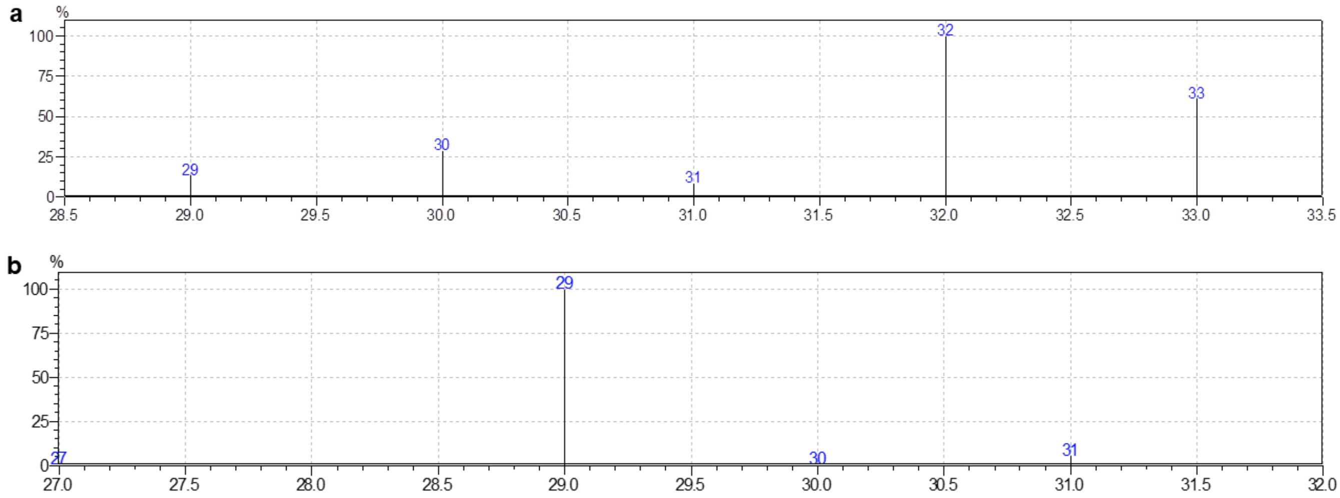

Supplementary Figure 8. GC-MS raw data: Carbon-13 isotopic labelling measurement of (a) methanol and (b) CO in the atmosphere of $^{13}CO_2$. Source data are provided as a Source Data file.

Supplementary Methods

Computational details

All the calculations were carried out using Vienna *ab-initio* simulation Package (VASP).[10] The projected augmented wave (PAW) potentials were adopted to treat the core electrons, and electron exchange and correlation were treated using by PBE functional.[11] The energy cutoff was set to 500 eV. All the atoms were fully relaxed until the total energies were converged up to 10^{-5} eV and the Hellmann-Feynman forces were less than 0.01 eV /Å. Only Gamma point was using for the k-point sampling. For the gas phase adsorption of CO_2 and CH_3OH, DFT-D2 method of Grimme was adopted for the van der Waals correction.[12] Spin polarizations were considered in the whole calculations.

2.1 CD model

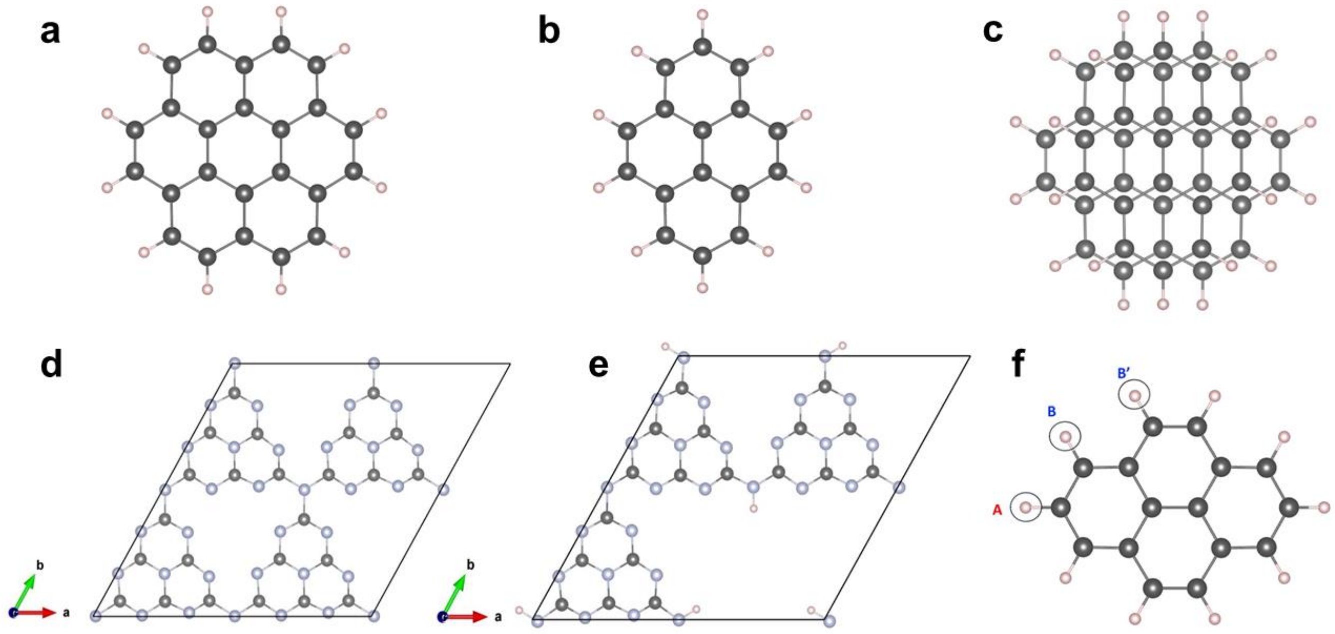

Supplementary Figure 9. The ^{m}CD was adopted from two carbon nanoflakes, (a) coronene and (b) pyrene, symmetrically stacking together. The grey and pink balls represent carbon and hydrogen atoms, respectively. (c) The top view of the ^{m}CD structure. It consists of a coronene at the bottom and a pyrene on top. (d) The top view of the structure of CN. The grey and purple balls represent carbon and nitrogen atoms, respectively. (e) The top view of protonated porous CN. (f) The denotations of the different adsorption sites for CO_2 and CH_3OH by the positions of the H atoms on ^{m}CD.

2.2 The adsorption energy

The adsorption energy, E_{ad}, was calculated according to

$$E_{ad}= E_{total}-(E_{adsorbate}+E_{adsorbent}) \qquad (4)$$

where E_{total}, $E_{adsorbate}$ and $E_{adsorbent}$ represent the energies of the absorbing system, the adsorbate and the adsorbent at equilibrium configurations, respectively. The results for the most stable configurations are listed in Supplementary Tables 3 to 5.

Supplementary Table 3. The adsorption energies, E_{ad} (in eV), of CO_2, CH_3OH and H_2O on ^{m}CD with different configurations.

Configurations	CO_2	CH_3OH	H_2O
A_o	-4.98E-02	-1.04E-01	-1.03E-01
A_c	-7.08E-02	-1.28E-01	-1.01E-01
A_p	-7.28E-02	-1.28E-01	-8.34E-02
B_o	-6.14E-02	-9.07E-02	-1.08E-01
B_c	-9.89E-02	-1.16E-01	-1.12E-01
B_p	-7.79E-02	-2.76E-01	-8.89E-02
AB	-1.12E-01	-1.60E-01	-2.29E-01
AB_p	-8.73E-02	-2.89E-01	-2.30E-01
BB	-1.07E-01	-1.49E-01	-1.16E-01
BB_p	-9.61E-02	-1.34E-01	-2.10E-01

Supplementary Note:

According to Supplementary Figure 10, ten different adsorption configurations were considered. They included along or perpendicular to the A or B hydrogen bonds denoted as A/B_o, A/B_c, respectively. There are also the bonding sites like along the A or B hydrogen bonds but perpendicular with the ^{m}CD plane denoted as A/B_p. More configurations included those parallel or perpendicular with AB and BB' directions. The adsorption energies results for different configurations were listed in Supplementary Table 3. Overall, the hydrogen bonds play an essential role in the strength of the adsorption. For CO_2, both AB and BB' sites are the top two stable sites, which involves forming two hydrogen bonds. Compared with CO_2, CH_3OH possesses stronger binding energy by a magnitude.

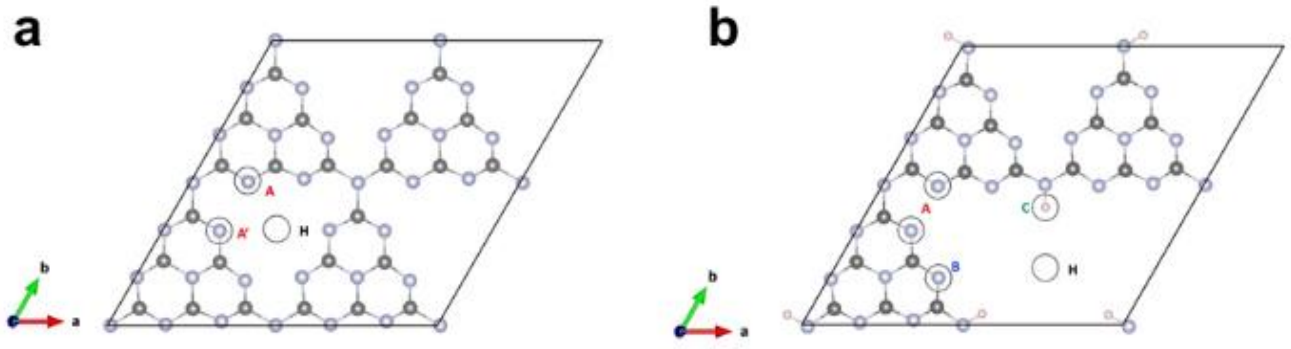

Supplementary Figure 10. (a) The denotations of distinct positions of CN for CO_2 adsorptions. (b) The denotations of distinct positions of protonated porous GCN for CO_2, CH_3OH and H_2O adsorptions.

Supplementary Table 4. The adsorption energies, E_{ad} (in eV), of CO_2 on CN with different configurations.

Configurations	CO_2
AA'	3.19E-03

AA'_p	2.64E-01
H	2.64E-01
H_p	8.70E-01

Supplementary Table 5. The adsorption energies, E_{ad} (in eV), of CO_2, CH_3OH and H_2O on protonated porous CN with different configurations.

Configurations	CO_2	CH_3OH	H_2O
AA'	-2.80E-01	-3.43E+00	-6.55E-2
AA'_p	-2.23E-01	-4.03E-01	N/A
AB	-1.69E-01	N/A	-6.66E-2
B	-2.31E-01	-3.82E+00	N/A
B_p	-1.79E-01	3.64E+00	N/A
C	-1.83E-01	-3.96E+00	-8.94E-2
C_p	-2.14E-01	-3.91E+00	N/A
H_p	-1.84E-01	-5.26E-01	N/A

Supplementary Note:

Similarly, several adsorption sites for CO_2 and CH_3OH on CN and protonated porous CN are also denoted, shown in Supplementary Figure 10, respectively. For CN, 4 possible configurations were selected, which were parallel or perpendicular with the nanosheet along AA' or at H positions, denoted as AA', AA'_p, H and H_p, respectively. For protonated porous CN, more possible configurations were considered, besides those in CN. They were parallel or perpendicular with AB, hydrogen bond at B and C, and H-the hollow position. The calculated adsorption energies of CO_2 and CH_3OH were listed in Supplementary Tables 4 and 5 for CN and protonated CN, respectively. However, due to the size of the CH_3OH molecule, it is unrealistic to calculate its adsorption energy on CN. Hence, the comparison of the CO_2 and CH_3OH is only carried out on the protonated porous CN.

Supplementary References

1 Xie, J. *et al.* Highly selective oxidation of methane to methanol at ambient conditions by titanium dioxide-supported iron species. *Nat. Catal.* **1**, 889-896, (2018).

2 Truong, Q. D., Le, T. H., Liu, J.-Y., Chung, C.-C. & Ling, Y.-C. Synthesis of TiO2 nanoparticles using novel titanium oxalate complex towards visible light-driven photocatalytic reduction of CO_2 to CH_3OH. *Appl. Catal., A* **437**, 28-35, (2012).

3 Wang, Z.-Y., Chou, H.-C., Wu, J. C. S., Tsai, D. P. & Mul, G. CO2 photoreduction using NiO/InTaO4 in optical-fiber reactor for renewable energy. *Appl. Catal., A* **380**, 172-177, (2010).

4 Mao, J. *et al.* Effect of graphitic carbon nitride microstructures on the activity and selectivity of photocatalytic CO2 reduction under visible light. *Catal. Sci. Technol.* **3**, 1253-1260, (2013).

5 Gusain, R., Kumar, P., Sharma, O. P., Jain, S. L. & Khatri, O. P. Reduced graphene oxide–CuO nanocomposites for photocatalytic conversion of CO2 into methanol under visible light irradiation. *Appl. Catal., B* **181**, 352-362, (2016).

6 Zhang, Q., Lin, C.-F., Chen, B.-Y., Ouyang, T. & Chang, C.-T. Deciphering Visible Light Photoreductive Conversion of CO2 to Formic Acid and Methanol Using Waste Prepared Material. *Environ. Sci. Technol.* **49**, 2405-2417, (2015).

7 Mao, J., Peng, T., Zhang, X., Li, K. & Zan, L. Selective methanol production from photocatalytic reduction of CO2 on BiVO4 under visible light irradiation. *Catal. Commun.* **28**, 38-41, (2012).

8 Li, A. *et al.* Adjusting the Reduction Potential of Electrons by Quantum Confinement for Selective Photoreduction of CO2 to Methanol. *Angew. Chem. Int. Ed.* **131**, 3844-3848, (2019).

9 Wang, L. *et al.* Photocatalytic Hydrogenation of Carbon Dioxide with High Selectivity to Methanol at Atmospheric Pressure. *Joule* **2**, 1369-1381, (2018).

10 Kresse, G. & Furthmüller, J. Efficient iterative schemes for ab initio total-energy calculations using a plane-wave basis set. *Phys. Rev. B* **54**, 11169-11186, (1996).

11 Perdew, J. P. *et al.* Atoms, molecules, solids, and surfaces: Applications of the generalized gradient approximation for exchange and correlation. *Phys. Rev. B* **46**, 6671-6687, (1992).

12 Grimme, S. Semiempirical GGA-type density functional constructed with a long-range dispersion correction. *J. Comput. Chem.* **27**, 1787-1799, (2006).
